# Diagnostic Potential of Selected Matrilysins and Stromelysins in the Diagnosis of Gynecological Malignancies Based on ROC Curve Analysis

**DOI:** 10.3390/ijms27125592

**Published:** 2026-06-20

**Authors:** Ewa Gacuta, Monika Zajkowska, Michał Ławicki, Julia Urban, Piotr Laudański, Monika Zbucka-Krętowska, Mateusz Antoni Józefczak, Tomasz Guszczyn, Paweł Ławicki, Marlena Dubatówka, Aleksandra Kicman, Sławomir Ławicki

**Affiliations:** 1 Department of Perinatology, University Clinical Hospital of Bialystok, 15-276 Białystok, Poland; sunnyeve@wp.pl; 2 Department of Neurodegeneration Diagnostics, Medical University of Białystok, 15-269 Białystok, Poland; monika.zajkowska@umb.edu.pl; 3 Department of Population Medicine and Lifestyle Diseases Prevention, University of Białystok, 15-269 Białystok, Poland; mlawicki@icloud.com (M.Ł.); juliaurban183@gmail.com (J.U.); pawellawicki04@gmail.com (P.Ł.); 4 Department of Obstetrics, Gynecology and Gynecological Oncology, Medical University of Warsaw, 03-242 Warsaw, Poland; piotr.laudanski@wum.edu.pl; 5 Women’s Health Research Institute, Calisia University, 62-800 Kalisz, Poland; 6 OVIklinika Infertility Center, 01-377 Warsaw, Poland; 7 Department of Gynecological Endocrinology and Adolescent Gynecology, Medical University of Białystok, ul. M. Skłodowskiej-Curie 24a, 15-276 Białystok, Poland; monikazbucka@wp.pl; 8 Department of Urology and Urological Oncology, Specialist Hospital in Puławy, 24-100 Puławy, Poland; mateusz.jozefczak@gmail.com; 9 Department of Pediatric Orthopaedics and Traumatology, Medical University of Białystok, 15-269 Białystok, Poland; tomasz.guszczyn@icloud.com; 10 Population Research Center, Medical University of Bialystok, 15-276 Bialystok, Poland; marlena.dubatowka@umb.edu.pl; 11 Department of Psychiatry, The Faculty of Medicine, Medical University of Bialystok, 15-269 Bialystok, Poland; olakicman@gmail.com

**Keywords:** MMP-3, MMP-7, MMP-10, MMP-26, diagnostic utility, ROC analysis

## Abstract

Matrilysins and stromelysins play a vital role in cancer, facilitating tumor invasion and metastasis. The aim of this study was to investigate the diagnostic significance of selected matrilysins and stromelysins in comparison to routine tumor markers in gynecological malignancies, relative to a control group (benign tumors and healthy women). Preoperative plasma levels of selected metalloproteinases were determined using ELISA, while levels of CA125, SCC-Ag, and HE4 by CMIA. In endometrial and cervical cancers, matrilysins (MMP-7 and MMP-26) exhibited higher diagnostic utility than routine markers. Similarly, all stromelysins in cervical cancer outperformed CA125; furthermore, MMP-10 also outperformed SCC-Ag, achieving the highest diagnostic utility among all parameters tested in cervical cancer. For ovarian cancer, diagnostic utility remained highest for routine markers. In endometrial and cervical cancers, the AUCs for all studied parameters exceeded those of standard markers, while in ovarian cancer, MMP-7 had an AUC higher than HE4 and comparable to CA 125. Combined analysis of the studied parameters in diagnostic panels demonstrated that their introduction into routine diagnostics could provide tangible benefits in the detection of malignant gynecological lesions, especially the combination of MMP-7 or MMP-10 with routine markers. These results indicate the usefulness and high diagnostic power of selected MMPs in the detection of these malignancies.

## 1. Introduction

Gynecological cancers, including, among others, cervical cancer (CC), ovarian cancer (OC), and endometrial cancer (EC), pose a significant health challenge. According to GLOBOCAN, in 2022, over 650,000 women died from these cancers—348,000 from cervical cancer, nearly 98,000 from endometrial cancer, and approximately 206,000 from ovarian cancer—accounting for more than 15% of all cancer deaths among women. In the same year, more than 660,000 new cases of cervical cancer, over 420,000 cases of endometrial cancer, and more than 324,000 cases of ovarian cancer were diagnosed, totaling over 1.4 million new cases. This represents 14.5% of all newly diagnosed cancers in women in 2022. In terms of incidence, these cancers rank 4th (CC), 6th (EC), and 8th (OC) among all cancers in women, and 4th, 8th, and 14th, respectively, in mortality rates among women globally [1,2].

Cervical cancer is most commonly caused by chronic infection with the human papillomavirus (HPV), particularly types 16 and 18. It ranks among the most prevalent cancers in women worldwide, especially in developing countries, where access to prevention methods (such as HPV vaccinations) and regular screening tests is often limited. HPV infection can cause the transformation of cervical epithelial cells, potentially leading to dysplasia and cancer. Additionally, factors such as chronic cervicitis, smoking, and immunosuppression can significantly accelerate the carcinogenic process. In developed countries, the incidence of cervical cancer has decreased due to screening programs (such as cytology); however, the number of newly identified cases remains concerning [3,4,5].

Ovarian cancer is a malignancy that arises from ovarian epithelial cells or tissues associated with the fallopian tubes. Characterized by its insidious onset, this type of cancer often progresses asymptomatically for extended periods, resulting in a late diagnosis frequently made at advanced stages [6]. Certain genetic mutations, e.g., *BRCA1* and *BRCA2* genes, have been identified as significant risk factors for the development of ovarian cancer. The incidence of this malignancy is particularly associated with the menopausal transition and the post-menopausal period, although it can also manifest in younger women. The diagnosis of ovarian cancer presents considerable challenges due to the nonspecific nature of its symptoms, which may include abdominal pain and bloating, often leading to confusion with benign conditions. Diagnostic modalities include ultrasonography (USG) and the assessment of serum levels of the cancer antigen 125 (CA 125). For the evaluation of advanced ovarian cancer, imaging techniques such as computed tomography (CT) and magnetic resonance imaging (MRI) play a critical role in determining disease extent and guiding treatment decisions [7,8].

Endometrial cancer predominantly arises from the endometrium, the mucosal lining of the uterus that undergoes cyclical transformations throughout the menstrual cycle. The pathogenesis of endometrial cancer is intricately linked to hormonal fluctuations, particularly an excess of estrogen relative to progesterone, which is a significant contributing factor to its development [9]. Furthermore, a substantial number of endometrial cancer cases are associated with mutations in genes that are pivotal to DNA repair mechanisms, such as those involved in the mismatch repair (MMR) pathway, which can lead to genomic instability. Additionally, alterations in key oncogenes and tumor suppressor genes, including *PTEN*, *PIK3CA*, and *KRAS*, have been implicated in the oncogenic process. The clinical presentation of endometrial cancer frequently includes abnormal uterine bleeding, particularly intermenstrual cycles or postmenopausal bleeding, which is recognized as the most prevalent symptom. Other potential manifestations include pelvic pain and dysuria, while ascites may occur in advanced stages of the disease. Diagnostic evaluation of endometrial cancer typically begins with a comprehensive gynecological examination. Complementary imaging modalities such as transvaginal ultrasonography (TVUS) and hysteroscopy are employed, with hysteroscopy allowing for direct visualization of the uterine cavity and enabling biopsy acquisition for histopathological analysis. This procedure is regarded as the gold standard for assessing pathological changes within the endometrium. Additionally, advanced imaging techniques such as CT and MRI play critical roles in staging the disease and evaluating potential metastatic spread to extrapelvic organs [9,10,11].

SCC-Ag for cervical cancer (specifically for squamous cell carcinoma—SCC), HE4 for ovarian cancer, and CA 125 for all three cancers are the key routine markers used in the laboratory diagnosis of gynecological cancers. However, it is important to note that elevated levels of CA 125 can occur in a variety of benign conditions, such as endometriosis, peritonitis, and ovarian cysts; thus, reliance on this marker alone requires careful consideration of clinical context and additional diagnostic information. This marker (similarly for SCC-Ag in SCC) is mainly used for monitoring the response to treatment (e.g., chemotherapy or radiotherapy) and detecting disease recurrence, especially in advanced stages of cancer. An additional biomarker utilized in the laboratory diagnosis of ovarian cancer—HE4 (human epididymis protein 4). Its levels may be significantly elevated in cases of ovarian cancer, particularly in the serous and endometrioid histological subtypes. The combination of HE4 and CA 125 facilitates the application of the ROMA (Risk of Ovarian Malignancy Algorithm) test, which enhances the accuracy of diagnostic assessments [8,12,13,14]. However, both the diagnostic sensitivity and specificity of the aforementioned tumor markers are not sufficient for inclusion in routine screening diagnostics; therefore, new markers with higher diagnostic utility are required.

Matrilysins and stromelysins are proteolytic enzymes that belong to the group of matrix metalloproteinases (MMPs). They play a crucial role in the degradation of the extracellular matrix (ECM). Their activity is essential for processes such as tissue remodeling, cell migration, invasion, and metastasis, which are characteristic of cancer progression, including gynecological cancers. These proteolytic enzymes facilitate the ability of cancer cells to penetrate the ECM and migrate to other tissues, which is a critical factor in the formation of metastases. It has been suggested that elevated expression of matrilysins and stromelysins may be associated with a more aggressive disease course and poorer prognosis [15,16,17].

Consequently, we decided to assess the potential of selected matrilysins (MMP-7 and MMP-26) and stromelysins (MMP-3, MMP-10) as prospective tumor biomarkers. Our objective was to determine whether the incorporation of these biomarkers into routine screening diagnostics could enhance the detection rates of gynecological cancers, thereby simplifying and accelerating the diagnostic process by limiting it to venous blood sampling.

## 2. Results

Table 1 shows diagnostic utility criteria (SE—sensitivity; SP—specificity; ACC—accuracy; PPV—positive predictive value; NPV—negative predictive value) for all tested parameters and routinely used tumor markers and their combinations in all three tumor types.

In the context of endometrial cancer, all diagnostic parameters obtained for MMP-7 and MMP-3 surpassed those of CA 125, with similar findings observed for MMP-26 and MMP-10, where only sensitivity exhibited a slightly lower value in comparison to the conventional biomarker. The combined analysis of both matrilysins/stromelysins and the routine marker did not significantly increase the diagnostic usefulness of the assessed panels.

For cervical cancer, the diagnostic sensitivity of all matrilysins and stromelysins was also superior to that of CA 125 and SCC-Ag, with analogous results noted for specificity (SP), accuracy (ACC), PPV, and NPV, although MMP-26 and MMP-3 presented comparable or slightly lower specificity. Combined analysis of both matrilysins and routine markers improved the diagnostic utility of the evaluated panels, achieving specificity, ACC, PPVs, and NPVs higher than those obtained with individual potential biomarkers. The highest values were achieved by the MMP-7 panel with CA125 and MMP-7 with both routine markers.

In the case of ovarian cancer, only the sensitivity of MMP-7 was higher than HE4, but lower than CA 125. The SP, ACC, NPV, and PPV for the studied parameters remained lower than those observed for the comparative markers. Combined analysis of both matrilysins and routine markers improved the diagnostic utility of the evaluated panels, achieving comparative or even higher sensitivity, specificity, ACC, PPV, and NPV than those obtained with individual potential biomarkers or routine markers. The highest values were achieved by the MMP-7 panel with CA125 and HE4.

The relationship between diagnostic sensitivity (SE) and specificity (SP) is exemplified by the Receiver Operating Characteristic (ROC) curve. The area under the ROC curve (AUC) serves as a metric for evaluating the clinical utility of a tumor marker, as well as its diagnostic efficacy. Furthermore, the AUC quantitatively assesses the overall capacity of the test to distinguish between individuals diagnosed with the disease and healthy control. Comprehensive data pertaining to the AUC values across different types of cancers are presented in Table 2.

**Figure 1:**
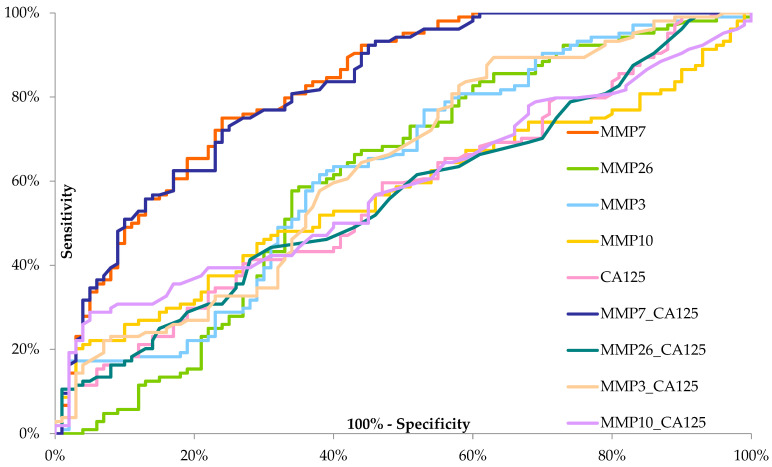
Graphical version of ROC curve analysis in Endometrial Cancer.

**Figure 2:**
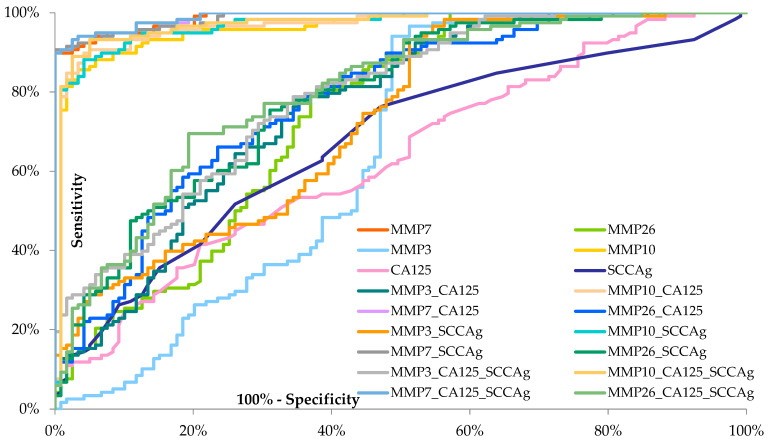
Graphical version of ROC curve analysis in Cervical Cancer.

**Figure 3:**
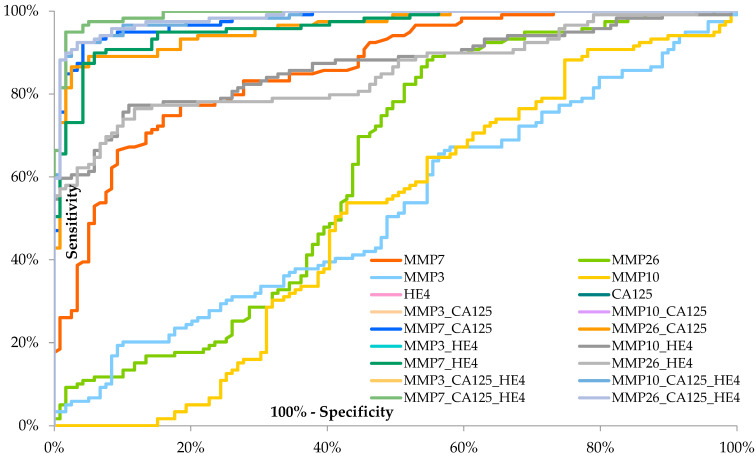
Graphical version of ROC curve analysis in Ovarian Cancer.

In the context of endometrial and cervical cancers, the area under the receiver operating characteristic (ROC) curve for almost all studied parameters exceeded that of the comparative markers (excluding MMP-10 in endometrial cancer). Conversely, in the case of ovarian cancer, only MMP-7 yielded an area under the curve (AUC) value higher than that obtained for HE4 and comparable to CA125. The combined analysis of the studied parameters with routine markers resulted in an increase in the AUC values for cervical and ovarian cancer. The most favorable result was obtained for the combination of MMP-7 with both comparative markers, reaching values above 0.99, which proves the extremely high usefulness of the proposed panel in detecting neoplastic lesions. Graphical representations of the ROC curves for all studied parameters, categorized by cancer types, are presented in Figure 1, Figure 2 and Figure 3.

## 3. Discussion

Matrilysins and stromelysins are proteolytic enzymes classified as matrix metalloproteinases (MMPs). These enzymes are crucial for the degradation of the extracellular matrix (ECM), a complex network of proteins and carbohydrates that provides structural and biochemical support to surrounding cells [15]. The activity of matrilysins and stromelysins is essential for various physiological processes, including tissue remodeling, cell migration, invasion, and metastasis. These processes are particularly significant in the context of cancer, where the ability of tumor cells to invade surrounding tissues and spread to distant sites is a key characteristic of malignancy. By breaking down the components of the extracellular matrix, these enzymes facilitate cellular motility, allowing them to migrate to new locations; this activity is vital during normal physiological processes such as wound healing and embryogenesis. However, in cancer, this same mechanism can contribute to the aggressive behavior of tumors, enabling them to detach from their primary site and establish secondary tumors in other organs [15,16,17]. Recent evidence highlights that tumor progression is not solely driven by cancer cells but is strongly regulated by the tumor microenvironment (TME), including stromal cells, immune infiltration, and extracellular matrix (ECM) remodeling. In particular, extracellular vesicles (EVs) have emerged as key mediators of intercellular communication within the TME, facilitating the transfer of proteins, RNAs, and regulatory molecules that promote epithelial–mesenchymal transition, angiogenesis, and metastatic niche formation. Importantly, EVs have been shown to carry active matrix metalloproteinases (including MMP-2, MMP-9, and MT1-MMP), directly contributing to ECM degradation and tumor invasion. Recent studies also indicate that EV-mediated signaling is closely linked to transcriptomic and post-transcriptional regulation, including alternative splicing events and non-coding RNA networks that modulate MMP expression in cancer cells. These mechanisms collectively underline the complexity of MMP regulation and their role in tumor progression beyond simple overexpression at the tissue level [18,19,20,21]. Several authors have confirmed that matrilysins and stromelysins can be produced by the cells of various malignant cell types, including, among others, colorectal cancer and breast cancer cells [22,23,24,25,26,27,28,29]. The expression and activity of MMPs are tightly regulated at multiple levels, including transcriptional control, epigenetic modifications, and post-translational activation. Moreover, their activity is modulated by tissue inhibitors of metalloproteinases (TIMPs) as well as by microenvironmental factors such as hypoxia and inflammatory cytokines. These regulatory layers may explain the context-dependent behavior of MMPs observed across different tumor types [30,31].

Overall, MMP activity in cancer reflects a complex interaction between tumor cells and the surrounding microenvironment rather than a simple overexpression phenomenon. Extracellular vesicle-mediated signaling, transcriptional reprogramming, and ECM–cell interactions collectively contribute to the regulation of MMP expression and activity, ultimately influencing tumor invasion, progression, and metastatic dissemination. In the context of endometrial (EC) and cervical (CC) cancers, the diagnostic usefulness of matrilysins (MMP-7 and MMP-26) identified through our analyses was significantly higher than that of the reference marker. All parameters of diagnostic utility (including sensitivity (SE), specificity (SP), accuracy (ACC), positive predictive value (PPV), negative predictive value (NPV), and area under the curve (AUC)) exhibited very high and statistically significant values, with the exception of the MMP-26 sensitivity in EC. These findings indicate a superior capacity of these parameters to differentiate between individuals with malignant lesions and healthy women or those diagnosed with benign endometrial/cervical changes. Furthermore, the CA125 marker did not yield statistically significant results in Endometrial Cancer (*p* = 0.07), suggesting its limited utility in detecting neoplastic transformations and raising concerns about its effectiveness in screening diagnostics. Given the critical importance of incorporating tests with the highest diagnostic utility for identifying ongoing neoplastic changes in screening protocols, our findings provide promising evidence for enhancing the detection rates. Interestingly, in the case of ovarian cancer, the newly studied parameters did not show higher usefulness than routine markers (with the exception of MMP-7). Regarding stromelysins, only MMP-10 in cervical and MMP-3 in endometrial cancer obtained a higher usefulness compared to routine markers. The evaluation of the proposed diagnostic panels showed that in the case of ovarian and cervical cancer, it provides measurable benefits, especially when combining MMP-7 with routine markers.

In the existing literature, we identified studies examining the presence of matrilysins and stromelysins in women diagnosed with gynecological cancers. However, most of these studies did not evaluate the diagnostic utility of these biomarkers. In the research conducted by Davidson et al. [32], a statistically significant elevation in the expression of this protein (MMP-7) was reported in tissue samples from women with ovarian, endometrial, and cervical cancers. Similar reports were also presented by other authors [33,34,35,36,37,38,39,40,41,42,43]. Moreover, Davidson et al. [32] established that there were statistically significant differences in MMP-7 expression levels between high-grade serous carcinoma (HGSC) and malignant mesothelioma. They estimated the sensitivity and specificity of MMP-7 to be 46% and 100%, respectively. Given that our study did not include patients with malignant mesothelioma, we are unable to reference or compare our findings to these statistics. The authors concluded that MMP-7 expression exhibits high specificity, although only moderate sensitivity, for the diagnosis of carcinoma, supporting its use in differentiating carcinoma from both benign and malignant mesothelial cells, which is partially consistent with our results. The authors observed that the expression of the studied marker was more prevalent in ovarian and other tumors of the female genital tract, specifically the cervix and endometrium, when compared to metastatic cancers originating from the breast, lung, or gastrointestinal tract. A comparative analysis focused on high-grade serous carcinoma (HGSC) and breast cancer similarly revealed a statistically significant difference in MMP-7 expression levels. Furthermore, among the various histological types of ovarian cancer, the expression was notably higher in clear cell carcinoma (87%) compared to both high-grade serous carcinoma (46%) and low-grade serous carcinoma (38%), which we regard as a significant finding [32]. Additionally, research conducted by Markova et al. [44] evaluated the immunohistochemical expression levels of MMP-7 and MMP-26 in a cohort of 70 endometrial cancer patients. The findings indicated that MMP-7 was positively expressed in 33 patients (47.1%), while MMP-26 was positively expressed in 40 patients (57.1%). Similar reports have been presented by other authors [45]. Notably, the study observed a decline in the expression of MMP-7 with increasing patient age.

In a separate study, Gershtein et al. [46] evaluated the concentrations of matrix metalloproteinase-7 (MMP-7) in patients diagnosed with endometrial cancer, those with benign lesions, and a cohort of healthy women. Their findings indicated that both carcinoma and benign neoplasms were associated with a significant elevation in MMP-7 levels. The analyses conducted yielded a cut-off point comparable to that reported in our study (3.5 ng/mL vs. 2.87 ng/mL, respectively). Furthermore, the authors performed an ROC analysis, which demonstrated a high sensitivity and specificity for MMP-7, reported at 88% and 87%, respectively. These results are largely consistent with our findings, which indicated sensitivity and specificity values of 76% and 75%, respectively. The observed discrepancy may be attributed to the composition of the control group. Gershtein et al. exclusively included healthy individuals, whereas our control group comprised women with benign lesions in addition to healthy subjects. Notably, the authors did not present additional data regarding the diagnostic utility of MMP-7. They concluded that MMP-7 was unable to differentiate between benign and malignant endometrial lesions, a finding that contrasts with our results. Our study demonstrates that the control group, which included both healthy women and those with benign lesions, exhibited significant differences in MMP-7 concentrations, thereby allowing for effective differentiation between malignant and benign lesions as well as healthy subjects with considerable accuracy.

In their study, Shiomi et al. [47] demonstrated that the concentration of matrix metalloproteinase-7 (MMP-7) in the supernatants of tissue homogenates from patients diagnosed with endometrial cancer was significantly elevated compared to the concentrations observed in supernatants derived from healthy tissue homogenates. Furthermore, they found that the levels of MMP-7 production exhibited a correlation with lymph node metastasis. Additionally, as described in the works of Ueno et al. [48] and Yamamoto et al. [49], immunoblotting and zymographic analyses revealed that proMMP-7 is efficiently activated within carcinoma tissues, suggesting that MMP-7 activity plays a role in the metastatic process. Collectively, these findings underscore the potential utility of this metalloproteinase as a biomarker in endometrial cancer, reflecting its significant involvement in tumor progression and the metastatic cascade.

In cervical cancer, Guo et al. [50] demonstrated that the expression of matrix metalloproteinase-7 (MMP-7) was significantly elevated in patients diagnosed with adenocarcinomas and adenosquamous carcinomas, as well as those exhibiting pelvic lymph node metastasis. Furthermore, the area under the receiver operating characteristic (ROC) curve for MMP-7 was reported to be 0.707. The statistically significant findings from Guo et al. corroborate our own research, which indicated an even greater predictive capacity for this biomarker in cervical cancer, with an AUC of 0.988. These discrepancies in results may be attributed to differences in the patient populations studied. Specifically, Guo et al. focused on individuals with early-stage cervical cancer (Stage Ia2-IIa2), while our cohort encompassed women across all stages of cancer progression. Additionally, the methodologies employed to assess the diagnostic utility of MMP-7 in identifying cervical cancer varied between the studies. Consequently, it may be asserted that both the concentration and expression levels of MMP-7 hold significant clinical utility, positioning this biomarker as a promising candidate for cervical cancer detection.

The aforementioned hypotheses are supported by the findings reported by Zhu et al. [51]. In their study, the authors conducted a comprehensive analysis of MMP-7 expression in cervical cancer tissues using quantitative real-time polymerase chain reaction (qRT-PCR) and Western blotting techniques. They employed gene silencing methods to investigate the functional role of MMP-7 in cervical cancer cells and assessed serum MMP-7 levels in patients diagnosed with cervical cancer compared to healthy controls through enzyme-linked immunosorbent assay (ELISA). The results indicated that both mRNA and protein levels of MMP-7 were significantly elevated in cervical cancer tissues relative to those in healthy controls. Additionally, the silencing of MMP-7 resulted in a marked reduction in cellular proliferation, migration, and invasion of cervical cancer cells. Notably, serum MMP-7 levels were significantly higher in patients with cervical cancer compared to healthy individuals. Furthermore, elevated MMP-7 expression was correlated with increased lymphatic metastasis, higher pathological grade, and advanced clinical stage of the disease. The described results are consistent with our results, contributing to the confirmation of the hypothesis regarding the high usefulness of MMP-7 in the diagnosis of cervical cancer.

In ovarian cancer, a number of studies have evaluated the diagnostic utility of MMP-7, enabling us to conduct relevant comparisons and analyze the results obtained. In the study conducted by Simmons et al. [52], the authors reported a sensitivity and specificity for MMP-7 of 49.2% and 82.8%, respectively, at a cut-off threshold of 2.06 ng/mL. In contrast, our analysis, utilizing a cut-off point determined by the Youden index at 3.23 ng/mL, yielded diagnostic sensitivity and specificity values of 77.3% and 81.5%, respectively. These discrepancies may be attributed to differences in the selection criteria for the control group, as the aforementioned study included only healthy postmenopausal women as controls. Moreover, the authors of the cited work conducted an additional analysis that involved the simultaneous assessment of multiple parameters within a four-marker panel comprising CA125, HE4, MMP-7, and CA72-4. This comprehensive approach resulted in a significant enhancement in the diagnostic efficacy for detecting neoplastic lesions in the ovaries, achieving a sensitivity of 83.2% at a specificity of 98%. This is one of the few studies that evaluates panels of potential biomarkers in a similar manner to ours. Although the composition of our proposed panel is slightly different (we did not study CA 72-4), our results also demonstrate that incorporating MMP-7 into a panel with routine markers offers significant benefits in terms of distinguishing malignant changes with high efficacy (SE: 94.96%; SP:98.32%).

A comparable analysis was also conducted by Będkowska et al. [53,54], where the diagnostic parameters were reported as follows: in the first study, SE = 61%, SP = 95%, PPV = 93%, NPV = 61%, AUC = 0.834; in the second study, the parameters were SE = 78%, SP = 84%, PPV = 72%, NPV = 88%, and AUC = 0.826. These findings align closely with our results, thereby providing strong evidence for the considerable diagnostic utility of MMP-7 in the detection of ovarian cancer. In both studies, the combination of CA125, HE4, and MMP-7 exhibited the highest diagnostic efficacy, resulting in an AUC of 0.938, which is consistent with our results (AUC = 0.992). Comparable findings were reported by Zohny et al. [55], who documented SE = 80.4%, SP = 87.5%, PPV = 85%, NPV = 83%, ACC = 84%, AUC = 0.907 for MMP-7 and the analysis with additional parameters showed an increase in diagnostic sensitivity. The multiparametric analysis undertaken by the authors demonstrated, in agreement with our work, that the simultaneous evaluation of multiple parameters, particularly when combined with currently utilized biomarkers in routine diagnostics, significantly enhances diagnostic capabilities.

In the study conducted by Acar et al. [56], the authors investigated the levels of MMP-7 in a cohort comprising 30 women diagnosed with ovarian cancer. Despite the limited sample size, the findings revealed that MMP-7 levels were significantly elevated in the ovarian cancer group compared to the healthy control group. Notably, the concentrations of MMP-7 were assessed 7–10 days post-surgery, and the analysis indicated a significant reduction in these levels relative to preoperative measurements. This observation supports previous assertions that cancer cells serve as a source of MMP-7. Furthermore, the authors found that patients with lymph node metastasis exhibited higher serum levels of MMP-7 in comparison to those without lymph node metastasis, highlighting a potential correlation between MMP-7 levels and metastatic disease progression.

The results obtained for MMP-26 in EC and CC demonstrated statistical significance; however, only sensitivity was lower in comparison to the conventional tumor marker in EC. Notably, the area under the curve (AUC) values for endometrial and cervical cancers exceeded those of standard tumor markers. Unfortunately, there are no studies available on MMP-26 in cervical cancer. Additionally, studies conducted by Isaka et al. [57] and Nishi et al. [58] found no statistically significant differences between healthy tissue and endometrial cancer. However, these discrepancies could be attributed to the relationship between the cycle phase and MMP-26 mRNA expression revealed in the work of Pilka et al. [59]. Authors reported that MMP-26 mRNA is specifically expressed in epithelial cells of the normal endometrium, and that expression increases during the early part of the cycle to a maximum at midcycle, and then rapidly decreases to low levels in the late part of the cycle [60]. Furthermore, Tunuguntla et al. [61] demonstrated that MMP-26 is expressed in both endometrial carcinomas and benign endometrial tissue across various stages of the menstrual cycle. Analysis of staining intensity revealed that endometrial carcinomas exhibited a significantly higher expression of MMP-26 compared to benign endometrial tissue from the postmenopausal period. However, this difference was not observed when comparing with tissue from the secretory phase of the menstrual cycle. The discrepancies observed between our findings and the mRNA expression levels reported in the literature are noteworthy. These differences may be attributed not only to the cycle phase dependency identified by the authors but also to the methodological approaches employed in their studies. It is important to note that while gene expression levels may exhibit considerable variability, the concentrations of previously isolated circulating proteins may remain relatively stable, potentially leading to divergent conclusions across studies. Furthermore, our research represents a significant contribution to the field, as it is the first to evaluate the concentrations and diagnostic utility of MMP-26 in the context of endometrial cancer. This novelty underscores the importance of our findings in the broader landscape of existing literature. However, a limitation of our study is the absence of data regarding the menstrual cycle phase during which blood samples were collected from patients. Incorporating this information could enhance the diagnostic utility parameters, particularly if variability in MMP-26 concentrations relative to the cycle phase is confirmed. Such an understanding could lead to more accurate diagnostic assessments in future research.

In the context of ovarian cancer, Zhao et al. [62] reported no statistically significant difference in the expression of MMP-26, a finding that contradicts our own observations. Conversely, the study conducted by Ripley et al. [63] demonstrated an elevated intensity of immunostaining for MMP-26 in invasive tumor cells, with expression levels increasing in correlation with tumor stage. The sole study assessing the diagnostic utility of MMP-26 concentration in ovarian cancer was conducted by Kicman et al. [64]. In this investigation, the authors reported a significant difference in MMP-26 concentrations between women diagnosed with ovarian cancer and healthy controls. The diagnostic parameters established in their study included a sensitivity (SE) of 78%, specificity (SP) of 68%, positive predictive value (PPV) of 85%, negative predictive value (NPV) of 57%, and an area under the curve (AUC) of 0.775. While these results were generally superior to those obtained in our analysis—excluding NPV—this discrepancy may be attributed to the composition of the control group, which consisted solely of healthy women without benign ovarian lesions. This aforementioned study represents the only comparable research to our findings, underlining the exceptionality of our investigation. These authors also performed an analysis similar to ours, combining the studied parameters with the ROMA algorithm, which, similarly to our work, provided benefits in higher diagnostic usefulness.

In our research, we have demonstrated that MMP-3 exhibits greater diagnostic utility in endometrial and cervical cancers compared to CA125 (although it is less effective than SCC-Ag in the case of cervical cancer). Conversely, its utility in ovarian cancer appears to be limited. Interestingly, studies by Liokumovich et al. [65] and Mannelqvist et al. [66] demonstrated that the MMP3 gene was significantly expressed in tumor cells and was associated with more aggressive and invasive EC tumors, showing more diffuse expression in high-grade than in low-grade tumors. Our previous studies on early-stage EC showed that plasma MMP-3 concentrations were significantly higher in patients with endometrial cancer compared to healthy women, but did not differ significantly from those in patients with benign lesions. Importantly, MMP-3 also did not demonstrate high sensitivity or specificity as a standalone marker [67]. To date, these are the only published studies on MMP-3 concentrations in EC.

Regrettably, our literature review revealed a lack of published studies addressing the utility of MMP-3 testing in the context of cervical cancer. This absence of relevant research hinders our ability to effectively compare the findings and unequivocally validate our results. However, the elevated expression of MMP-3 in cervical cancer has previously been documented by Davidson et al. [68]. Additionally, Argüello-Ramírez et al. [69] reported increased levels of MMP-3 in tumor explants derived from cervical cancer patients. Their study employed zymography and densitometric quantification, revealing a correlation between MMP-3 levels and resistance to radiotherapy, suggesting that MMP-3 may serve as a prognostic marker for poor outcomes in cervical cancer. Notably, a cross-analysis of The Cancer Genome Atlas (TCGA) database and the MetazSecKB database, conducted by Shao et al. [70], indicated that the MMP3 gene was significantly upregulated in cervical cancer patients. Furthermore, they found that MMP3 protein levels were markedly elevated in the serum of clinical cervical cancer patients, with a noticeable decrease following treatment. Moreover, these published data suggest not only a substantial diagnostic utility for MMP-3 in cervical cancer but also its potential relevance in guiding anticancer therapy. This assertion is supported by the work of Sato et al. [71], who investigated EMMPRIN and reported that the addition of this inducer led to increased expression of MMP-3 in vitro, which was associated with accelerated cancer cell development compared to cell lines lacking EMMPRIN. Similarly, Hsiao et al. [72] demonstrated significant suppression of invasion and migration through the downregulation of MMP-3 by Phloretin.

In the context of ovarian cancer, a study by Kicman et al. [64] investigated the diagnostic utility of matrix metalloproteinase-3 (MMP-3). The authors reported that MMP-3 concentrations were elevated in the plasma of individuals with both malignant and benign ovarian lesions. They established the diagnostic performance parameters for MMP-3, which were as follows: sensitivity (SE) = 64%, specificity (SP) = 68%, positive predictive value (PPV) = 83%, negative predictive value (NPV) = 44%, and an area under the curve (AUC) = 0.745. The discrepancies observed in our study, which did not reach statistical significance, may be attributed to the composition of the control group, which included healthy individuals as well as those with benign lesions. This finding suggests that MMP-3 may be a useful biomarker for differentiating healthy individuals from those with neoplastic lesions, although it does not provide the ability to discern between benign and malignant conditions. Additionally, the authors performed a supplementary analysis that incorporated multi-parameter panels, demonstrating that the combination of MMP-3 measurements with other metalloproteinases and routine biomarkers significantly enhances the diagnostic efficacy for identifying neoplastic lesions in the ovary [64], which confirms our reports. Similar comparative analyses were conducted by Cymbaluk-Płoska et al. [73], who reported sensitivity and specificity values for MMP-3 at 66% and 69%, respectively, with an area under the curve (AUC) of 0.753. Although these values were higher than those obtained in our study, they also did not surpass the performance of CA 125 or HE4. Furthermore, the same authors extended their research on MMP-3, demonstrating a correlation between this biomarker and overall survival in ovarian cancer patients [74]. The overexpression of the MMP-3 gene has been observed by other authors, including in animal models [75]. High levels of MMP-3 protein in tumors have also been identified as a significant negative prognostic indicator for patients [76]. Interestingly, inhibitors of MMP-3 have demonstrated efficacy as tumor suppressors in ovarian cancer [77]. Therefore, it is suggested to continue research on the usefulness of MMP-3 in ovarian cancer.

In the context of MMP-10, our research has established its significant utility as a biomarker in cervical cancer. However, its relevance in endometrial and ovarian cancer remains unsubstantiated. Notably, in cervical cancer, MMP-10 has demonstrated remarkable diagnostic efficacy, exhibiting an SE of 89.83%, SP of 91.60%, PPV of 91.38%, NPV of 90.08%, overall accuracy of 90.72%, and an AUC of 0.963, thus outperforming conventional tumor markers utilized in standard diagnostic practices. Unfortunately, there is a paucity of literature that allows for a direct comparison with our findings. Nevertheless, the work of Zhang et al. [78] provides valuable insights, as the authors utilized immunohistochemical techniques to illustrate that MMP-10 may play a crucial role in tumor growth and progression, suggesting that modulation of MMP-10 could represent a viable therapeutic strategy in oncology. In the case of ovarian cancer, only two studies have addressed the potential role of MMP-10. Wei et al. [79] demonstrated that MMP-10 expression is significantly elevated in cancerous tissues relative to healthy tissues, with MicroRNA-205 identified as a factor that reduces its expression. Conversely, Ke et al. [80] indicated that elevated MMP-10 levels may be attributed to tumor-associated macrophages (TAMs) that originate from peripheral blood monocytes and are recruited to the tumor microenvironment, where they exhibit functions conducive to tumor progression. Given the limited number of studies investigating MMP-10 in both cervical and ovarian cancers, further research is imperative to elucidate the potential of this biomarker in the detection and treatment of gynecological malignancies.

Although the cut-off analysis identified lower circulating MMP-3 concentrations in cervical cancer and lower MMP-10 concentrations in ovarian cancer relative to the control group, these findings should be interpreted with caution. The control group in the present study comprised not only healthy individuals but also patients with benign gynecological lesions. Notably, concentrations of these metalloproteinases were higher in patients with benign conditions than in healthy controls, which may have influenced the calculated cut-off values and the direction of discrimination observed in ROC analyses. Therefore, the observed pattern does not necessarily indicate biological downregulation of MMP-3 or MMP-10 in malignant disease but may rather reflect the heterogeneity of the control population and the known involvement of matrix metalloproteinases in inflammatory and tissue-remodeling processes associated with benign gynecological disorders. This observation further emphasizes the complexity of interpreting circulating MMP concentrations and highlights the need for future studies evaluating these biomarkers separately in healthy controls, benign lesions, and malignant tumors.

The evaluation of diagnostic panels appears to play a crucial role in improving the early detection and characterization of gynecological malignancies. Incorporating such panels into routine diagnostic practice could significantly enhance the accuracy and timeliness of cancer detection, ultimately benefiting patient outcomes. However, there are relatively few studies addressing this topic, which limits broader discussion and the implementation of these approaches. In our study, the use of diagnostic panels combining MMP-7 with routine tumor markers in ovarian and cervical cancers, as well as MMP-10 with standard markers in cervical cancer, has yielded promising results. These findings suggest that integrating selected matrix metalloproteinases with established biomarkers may improve diagnostic performance and warrants further investigation in larger clinical settings. However, although the present findings are promising, further studies involving external validation cohorts and multivariable analyses adjusted for clinicopathological characteristics are required to strengthen the translational and clinical relevance of the results. Such analyses would allow assessment of the independent diagnostic value of the investigated biomarkers and improve their potential applicability in clinical practice.

## 4. Materials and Methods

### 4.1. Patients

The study involved 360 women diagnosed by a team of oncologists with cervical, endometrial, and ovarian cancers. All patients were treated at the Department of Gynecology and the University Oncology Center at the University Clinical Hospital in Bialystok, Poland. Tumor classification and staging were conducted in accordance with the International Federation of Gynecology and Obstetrics (FIGO) classification. Histopathological diagnoses were established in all cases through tissue biopsy or surgical resection of tumor samples. Pretreatment staging procedures included physical examinations, blood tests, ultrasound scans, and X-rays. In accordance with worldwide clinical guidelines, all patients had computed tomography (CT) and magnetic resonance imaging (MRI) of the pelvic organs. Women with renal failure were excluded from the trial as they revealed high levels of HE4. None of the patients had received chemotherapy or radiotherapy prior to blood sample collection. A subset of these patients was previously included in our pilot study.

In this study, we recruited 60 healthy volunteers and 180 women with benign tumors (60 with each benign change) who underwent gynecological examinations before blood sample collection, to create a diverse control group, facilitating the identification of malignant lesions. Histopathological diagnoses for benign tumors were made in all cases through tissue biopsy or after surgical intervention. The study was approved by the local Ethics Committee at the Medical University of Bialystok (committee approval numbers: R-I-002/314/2009; R-I-002/239/2014; APK.002.420.2021), and all patients provided informed consent for participation in the study. Table 3 shows the data summary for the study’s patient and control groups.

### 4.2. Biochemical and Statistical Analysis

Venous blood samples were collected from each patient, centrifuged to obtain plasma, and stored at −85 °C until analysis. The plasma levels of the tested parameters were measured using an enzyme-linked immunosorbent assay (ELISA) in accordance with the manufacturer’s protocols (R&D Systems, Minneapolis, MN, USA, ElabScience, Wuhan, China, and Abbkine, Atlanta, GA, USA). Duplicate samples were assessed for each standard, control, and test sample. Routine markers were measured using chemiluminescent microparticle immunoassay (CMIA), in accordance with the manufacturer’s guidelines (Roche Diagnostics GmbH, Mannheim, Germany).

The statistical analysis was carried out using STATISTICA 14.0. We created a receiver operating characteristic (ROC) curve for all examined parameters and routine markers. The areas under the ROC curve (AUCs) were determined to evaluate diagnostic accuracy and compare AUCs for all evaluated factors, both individually and in combination with routinely used tumor markers. Statistically significant was defined as *p* < 0.05. The cut-off values were established using Youden’s index as the criterion for setting the optimal cut-off point. Table 4 shows all selected optimal cut-off points for the tested parameters and routine markers.

## 5. Conclusions

Currently, researchers are investigating biomarkers that facilitate the rapid identification of ongoing neoplastic processes. Given that matrilysins and stromielysins play a significant role in both physiological and pathological processes, including tumor invasion and metastasis, we aimed to evaluate their potential utility in detecting gynecological cancer lesions. Our findings demonstrate a notable diagnostic value for both matrilysins and stromielysins, particularly in the context of endometrial and cervical cancers. Specifically, we observed that MMP-7 exhibited the highest diagnostic utility in endometrial cancer, while both MMP-7 and MMP-10 showed substantial diagnostic performance in cervical cancer. In the case of ovarian cancer, the diagnostic parameters assessed did not surpass the efficacy of conventional markers. However, MMP-7 displayed results comparable to those of HE4. These findings underscore the promising diagnostic capabilities of selected MMPs in the detection of cervical and endometrial malignancies. Importantly, the combined analysis of the studied parameters together with routine markers in diagnostic panels proved that their introduction into routine diagnostics could provide tangible benefits in the detection of malignant gynecological lesions. Nevertheless, further research is necessary to comprehensively elucidate their potential in clinical practice.

## Figures and Tables

**Table 1:** Diagnostic utility criteria for tested parameters.

	Parameter	SE	SP	ACC	PPV	NPV
Endometrial Cancer	MMP-7	76.67%	75.00%	75.83%	75.41%	76.27%
	MMP-26	55.83%	67.50%	61.67%	63.21%	60.45%
	MMP-3	61.54%	62.00%	61.76%	62.75%	60.78%
	MMP-10	48.08%	68.00%	57.84%	60.98%	55.74%
	CA 125	60.00%	52.50%	56.25%	55.81%	56.76%
	MMP-7 + CA 125	75.83%	74.17%	75.00%	74.59%	75.42%
	MMP-26 + CA 125	45.00%	67.50%	56.25%	58.06%	55.10%
	MMP-3 + CA 125	64.42%	56.00%	60.29%	60.36%	60.22%
	MMP-10 + CA 125	56.73%	54.00%	55.39%	56.19%	54.55%
Cervical Cancer	MMP-7	94.07%	94.12%	94.09%	94.07%	94.12%
	MMP-26	78.81%	63.03%	70.89%	67.88%	75.00%
	MMP-3	94.07%	51.26%	72.57%	65.68%	89.71%
	MMP-10	89.83%	91.60%	90.72%	91.38%	90.08%
	SCC-Ag	76.27%	52.94%	64.56%	61.64%	69.23%
	CA 125	53.39%	64.71%	59.07%	60.00%	58.33%
	MMP-7 + SCC-Ag	92.37%	96.64%	94.51%	96.46%	92.74%
	MMP-26 + SCC-Ag	75.42%	68.91%	72.15%	70.63%	73.87%
	MMP-3 + SCC-Ag	74.58%	55.46%	64.98%	62.41%	68.75%
	MMP-10 + SCC-Ag	89.83%	93.28%	91.56%	92.98%	90.24%
	MMP-7 + CA 125	94.07%	96.64%	95.36%	96.52%	94.26%
	MMP-26 + CA 125	66.10%	76.47%	71.31%	73.58%	69.47%
	MMP-3 + CA 125	76.27%	65.55%	70.89%	68.70%	73.58%
	MMP-10 + CA 125	90.68%	94.96%	92.83%	94.69%	91.13%
	MMP-7 + SCC-Ag + CA 125	94.07%	96.64%	95.36%	96.52%	94.26%
	MMP-26 + SCC-Ag + CA 125	69.49%	80.67%	75.11%	78.10%	72.73%
	MMP-3 + SCC-Ag +CA 125	78.81%	65.55%	72.15%	69.40%	75.73%
	MMP-10 + SCC-Ag + CA 125	93.22%	94.96%	94.09%	94.83%	93.39%
Ovarian Cancer	MMP-7	77.31%	81.51%	79.41%	80.70%	78.23%
	MMP-26	69.75%	55.46%	62.61%	61.03%	64.71%
	MMP-3	65.55%	43.70%	55.04%	66.67%	55.91%
	MMP-10	53.78%	57.14%	55.46%	55.65%	55.28%
	HE4	76.47%	88.24%	82.35%	86.67%	78.95%
	CA 125	89.08%	94.96%	92.02%	94.64%	89.68%
	MMP-7 + HE4	89.92%	94.12%	92.02%	93.86%	90.32%
	MMP-26 + HE4	76.47%	88.24%	82.35%	86.67%	78.95%
	MMP-3 + HE4	76.47%	88.24%	82.35%	86.67%	78.95%
	MMP-10 + HE4	77.31%	89.08%	83.19%	87.62%	79.70%
	MMP-7 + CA 125	92.44%	95.80%	94.12%	95.65%	92.68%
	MMP-26 + CA 125	89.08%	94.96%	92.02%	94.64%	89.68%
	MMP-3 + CA 125	89.08%	94.96%	92.02%	94.64%	89.68%
	MMP-10 + CA 125	89.08%	94.96%	92.02%	94.64%	89.68%
	MMP-7 + HE4 + CA 125	94.96%	98.32%	96.64%	98.26%	95.12%
	MMP-26 + HE4 + CA 125	92.44%	96.64%	94.54%	96.49%	92.74%
	MMP-3 + HE4 + CA 125	92.44%	96.64%	94.54%	96.49%	92.74%
	MMP-10 + HE4 + CA 125	92.44%	96.64%	94.54%	96.49%	92.74%

Abbreviations: SE—sensitivity; SP—specificity; ACC—accuracy; PPV—positive predictive value; NPV—negative predictive value.

**Table 2:** Diagnostic criteria of ROC curve analysis for tested parameters.

	Parameter	AUC	SE	95% C.I. AUC	*p*
Endometrial Cancer	MMP-7	0.823	0.026	0.771–0.875	<0.001
	MMP-26	0.614	0.037	0.542–0.686	0.002
	MMP-3	0.625	0.039	0.548–0.702	0.002
	MMP-10	0.566	0.041	0.487–0.646	0.103
	CA 125	0.567	0.037	0.494–0.639	0.070
	MMP-7 + CA 125	0.820	0.027	0.768–0.872	<0.001
	MMP-26 + CA 125	0.560	0.037	0.487–0.633	0.105
	MMP-3 + CA 125	0.632	0.039	0.556–0.709	<0.001
	MMP-10 + CA 125	0.587	0.040	0.508–0.665	0.030
Cervical Cancer	MMP-7	0.988	0.005	0.979–0.997	<0.001
	MMP-26	0.737	0.033	0.673–0.801	<0.001
	MMP-3	0.642	0.038	0.568–0.717	<0.001
	MMP-10	0.963	0.012	0.939–0.986	<0.001
	SCC-Ag	0.672	0.035	0.603–0.740	<0.001
	CA 125	0.631	0.036	0.561–0.702	<0.001
	MMP-7 + SCC-Ag	0.988	0.005	0.978–0.997	<0.001
	MMP-26 + SCC-Ag	0.786	0.029	0.729–0.843	<0.001
	MMP-3 + SCC-Ag	0.724	0.033	0.660–0.788	<0.001
	MMP-10 + SCC-Ag	0.968	0.011	0.947–0.990	<0.001
	MMP-7 + CA 125	0.990	0.004	0.983–0.998	<0.001
	MMP-26 + CA 125	0.776	0.030	0.718–0.835	<0.001
	MMP-3 + CA 125	0.759	0.031	0.698–0.820	<0.001
	MMP-10 + CA 125	0.968	0.011	0.946–0.990	<0.001
	MMP-7 + SCC-Ag + CA 125	0.991	0.004	0.983–0.998	<0.001
	MMP-26 + SCC-Ag + CA 125	0.801	0.028	0.745–0.856	<0.001
	MMP-3 + SCC-Ag + CA 125	0.788	0.029	0.732–0.844	<0.001
	MMP-10 + SCC-Ag + CA 125	0.972	0.011	0.951–0.993	<0.001
Ovarian Cancer	MMP-7	0.862	0.023	0.817–0.908	<0.001
	MMP-26	0.619	0.038	0.546–0.693	0.002
	MMP-3	0.524	0.038	0.450–0.597	0.531
	MMP-10	0.501	0.039	0.425–0.576	0.983
	HE4	0.852	0.025	0.802–0.902	<0.001
	CA 125	0.961	0.011	0.938–0.983	<0.001
	MMP-7 + HE4	0.962	0.011	0.940–0.983	<0.001
	MMP-26 + HE4	0.852	0.025	0.802–0.902	<0.001
	MMP-3 + HE4	0.852	0.025	0.802–0.902	<0.001
	MMP-10 + HE4	0.868	0.024	0.821–0.916	<0.001
	MMP-7 + CA 125	0.978	0.008	0.963–0.993	<0.001
	MMP-26 + CA 125	0.961	0.011	0.938–0.983	<0.001
	MMP-3 + CA 125	0.961	0.011	0.938–0.983	<0.001
	MMP-10 + CA 125	0.961	0.011	0.938–0.983	<0.001
	MMP-7 + HE4 + CA 125	0.992	0.004	0.984–1.000	<0.001
	MMP-26 + HE4 + CA 125	0.985	0.006	0.973–0.996	<0.001
	MMP-3 + HE4 + CA 125	0.983	0.006	0.971–0.996	<0.001
	MMP-10 + HE4 + CA 125	0.983	0.006	0.971–0.996	<0.001

*p*—statistically significantly larger AUCs compared to AUC = 0.5; AUC—Area Under the Receiver Operating Characteristics (ROC) Curve; SE—standard error; C.I.—confidence interval.

**Table 3:** Characteristics of patients included in the study and control groups.

	Number of Patients	Median Age (Range)
**Study Group**		
**Endometrial Cancer**	120	59 (51–71)
**Cervical Cancer**	120	46 (25–61)
**Ovarian Cancer**	120	60 (22–81)
**Control Group**		
**Benign endometrial lesions** **(Myoma uteri)**	60	56 (49–70)
**Benign cervical lesions** **(Cervical ectropion)**	60	46 (23–58)
**Benign ovarian lesions** **(** **Serous cystadenomas)**	60	49 (16–80)
**Healthy women**	60	55 (48–69)

**Table 4:** Cut-off values for all tested parameters and routinely used tumor markers.

	Parameter	Selected Cut-Off Point
Endometrial Cancer	MMP-7	>2.87 ng/mL
	MMP-26	>9.51 ng/mL
	MMP-3	>9.38 ng/mL
	MMP-10	>854.55 ng/mL
	CA 125	>18.65 U/mL
Cervical Cancer	MMP-7	>202.35 ng/mL
	MMP-26	>7.04 ng/mL
	MMP-3	<20.25 ng/mL
	MMP-10	>759.97 ng/mL
	SCC-Ag	>0.85 U/mL
	CA 125	>17.85 U/mL
Ovarian Cancer	MMP-7	>3.23 ng/mL
	MMP-26	>8680.00 pg/mL
	MMP-3	>8676.64 pg/mL
	MMP-10	<98.40 pg/mL
	HE4	>67.11 U/mL
	CA 125	>50.60 U/mL

## Data Availability

The data presented in this study are available on request from the corresponding author.

## Abbreviations

The following abbreviations are used in this manuscript:

ACC	Accuracy
AUC	Area under the curve
BRCA1	Breast cancer type 1 susceptibility protein
BRCA2	Breast cancer type 2 susceptibility protein
CA125	Cancer Antigen 125
CC	Cervical Cancer
CI	Confidence interval
CMIA	Chemiluminescent Microparticle Immunoassay
CT	Computed tomography
DNA	Deoxyribonucleic acid
EC	Endometrial cancer
ECM	Extracellular matrix
ELISA	Enzyme-Linked Immunosorbent Assay
EMMPRIN	Extracellular Matrix Metalloproteinase Inducer
GLOBOCAN	Global Cancer Observatory
HE4	Human Epididymis Protein 4
HGSC	high-grade serous carcinoma
HPV	Human papillomavirus
KRAS	Kirsten rat sarcoma virus oncogene homolog
MMP-10	Matrix metalloproteinase 10
MMP-26	Matrix metalloproteinase 26
MMP-3	Matrix metalloproteinase 3
MMP-7	Matrix metalloproteinase 7
MMPs	Extracelluar Matrix Metalloproteinases
MMR	Mismatch Repair Genes
MRI	Magnetic resonance imaging
NPV	Negative predictive value
OC	Ovarian cancer
PPV	Positive predictive value
PTEN	Phosphatase and tensin homolog
ROC	Receiver Operating Characteristic
ROMA	Risk of Ovarian Malignancy Algorithm
SCC	Squamous cell carcinoma
SCC-Ag	Squamous cell carcinoma antigen
SE	Sensitivity
SP	Specificity
TVUS	Transvaginal ultrasonography
USG	Ultrasonography
